# Perceived Covid-19-crisis intensity and family supportive organizational perceptions as antecedents of parental burnout: A study conducted in Italy in March/April 2021 and 2022

**DOI:** 10.3389/fpsyg.2022.1001076

**Published:** 2022-10-06

**Authors:** Marta Redaelli, Marloes L. van Engen, Stéfanie André

**Affiliations:** ^1^Department of Human Resource Studies, Tilburg University, Tilburg, Netherlands; ^2^Department of Business Administration, Radboud University, Nijmegen, Netherlands; ^3^Radboud WORKLIFE consortium, Nijmegen, Netherlands; ^4^Department of Public Administration, Radboud University, Nijmegen, Netherlands

**Keywords:** work–family conflict, Covid-19 vaccination passport, perceived Covid-19 crisis intensity, parental burnout, family supportive organizational perceptions

## Abstract

The purpose of this study is to investigate to what extent perceived Covid-19-crisis intensity (PCCI) leads to the experience of parental burnout (PB), a syndrome characterized by exhaustion, emotional detachment from one’s own children and a sense of inefficacy in the role as parent. Furthermore, the mediating role of work–family conflict (WFC) is examined. The buffering effect of family supportive organizational perceptions during the pandemic (FSOP-p) on the relationship between work–family conflict and parental burnout is also explored. Data were collected in March–April 2021 and March/April 2022. In spring 2021, 222 Italian working parents with at least one minor child living at home filled out the questionnaire. Data from 2021 showed that PCCI was positively related to the experience of parental burnout. Moreover, WFC mediated this relationship. No significant interaction effect was found for FSOP-p; however it was found that FSOP-p is negatively related to PCCI and WFC, and indirectly to parental burnout. In spring 2022, we examined whether there were changes in PCCI, WFC, and FSOP-p in a sample of 83 Italian parents. Moreover, for the second data collection we examine the tensions experienced by parents in their families about vaccination and infection precaution measures (e.g., Covid-19 vaccination passport). The results are different in 2022; the effect of PCCI on parental burnout is now completely mediated by the amount of WFC. It seems that now we go ‘back to normal’ and homeworking has become more optional for many, there is still an effect of PCCI on WFC, but no longer directly on parental burnout. Furthermore, the prevalence of PCCI in 2022 is lower than in 2021, while WFC and FSOP-p are not significantly different between the two timepoints. As family supportive organizational perceptions reduce the level of perceived Covid-19 intensity, organizations are urged to develop practices of support and to create a supportive environment.

## Introduction

Ever since its outbreak early Spring 2020, the Covid-19 pandemic has not only caused an increasing number of infections and fatalities, but also enormous changes to the world of work and daily life (Banerjee and Rai, 2020). Governments and companies were urged by the World Health Organization to take drastic measures to prevent or contain the spread of the disease. During the course of the pandemic, containment measures imposed by governments varied in intensity, often intensifying following new surges in infection rates due to new Covid-19 variants, and lessening when infection rates or hospital intakes were decreasing. Measures ranged from strict lockdowns, closing of schools and leisure centers and cancellation of social activities, to ‘opening up society’ with entry requirements (e.g., QR entry codes, Covid-19 vaccination passports, admission requirements for public spaces). The latter solutions allowed more freedom of movement for those that are vaccinated or recovered from a Covid-19 infection, but less for others, for instance for the unvaccinated or people with health issues. This has led to new tensions in society that sometimes trickled down into the family sphere (Swit and Breen, 2022).

Furthermore, a great amount of the workforce has seen a transformation in their work dispositions (Rudolph et al., 2020). Especially for knowledge workers, the Covid-19 pandemic has forced a quick shift to (mandatory) full-time remote work, limiting the possibility to seek alternative workspaces other than one’s home (Carnevale and Hatak, 2020; Ghislieri et al., 2021). Such arrangements may have potential dramatic psychological impact on employees. First, due to the lack of familiarity with homeworking, employees may have experienced feelings of confusion and unclarity around how to organize and prioritize one’s tasks, how to use certain technological tools, whom to ask for support, how to approach colleagues or how to deal with new tasks (Bick et al., 2020; International Labour Office, 2020; Ghislieri et al., 2021). According to role theory (Roy et al., 1965), being constantly exposed to incongruent or vague expectations leads to role conflict and role ambiguity, conditions that may easily result in stress and anxiety (House and Rizzo, 1972). Second, working remotely generally requires the sustained use of technological devices, internet, emails and instant messaging and it usually implies multitasking, frequent system upgrades, recurring technical problems, continual relearning and consequent insecurities around tools and programs. These elements have been shown to induce technostress, which entails negative psychological states such as anxiety, irritability, overload, inability to switch off and burnout (Kolakowski, 2020; Molino et al., 2020; Spagnoli et al., 2020; Galanti et al., 2021; Ghislieri et al., 2021). Other psychological effects of working in confinement may entail employee isolation - a psychological construct that refers to employees’ perception of lacking opportunities for professional, social and emotional exchange with their co-workers (Brooks et al., 2020) – and a general sense of loneliness, also enhanced by regulations implying restriction of mobility, interruption of social and leisure activities, separation from loved ones, loss of freedom, lack of information and social support (Banerjee and Rai, 2020; Saladino et al., 2020). Long periods of isolation or quarantine have detrimental effects on mental wellbeing, including exhaustion, detachment from others, anxiety, irritability, insomnia, poor concentration and indecisiveness, deteriorating work performance, reluctance to work and consideration of resignation (Bai et al., 2004; Stickley and Koyanagi, 2016).

The purpose of this research is to examine how the transformations related to Covid-19, in terms of work pressure and social isolation, impacted a particularly vulnerable category: parents. The reasons for parents to be a population at risk during the pandemic regard their concerns about the physical and economic health of their family, about their ability to inform and reassure their children about Covid-19, about the outcomes of their children’s isolation and homeschooling, and about the often-unsupported management of both family and work demands (Fontanesi et al., 2020; Yerkes et al., 2020). In fact, while the abrupt modification in work arrangements caused an increase in work stress, the closure of schools and care facilities often demanded parents to simultaneously handle homeschooling and extra childcare responsibilities (Carnevale and Hatak, 2020). The experience of incompatible pressure arising at the same time in the work and in the family domain, called work–family conflict (Greenhaus and Beutell, 1985), is a further challenge with which parents had to cope during the pandemic (Caligiuri et al., 2020). The struggle to achieve balance between the work and family sphere, which both exerted higher demands than usual, may have led to negative health-related outcomes. This study focuses on parental burnout, a psychological and physical condition affecting those parents who experience a collapse in the ability to cope with chronic, overwhelming stress related to parenting (Mikolajczak et al., 2018). This syndrome is distinct, although parallel, to the syndrome of job burnout, and it is characterized by exhaustion, emotional detachment from one’s children and a deep sense of inefficacy in the role as parent (Roskam et al., 2017). Parental burnout has been found to associate with various outcomes such as depressive symptoms, sleep disorders, addictive behaviors, conflicts with the partner, escape ideation and child neglect or abuse (Kawamoto et al., 2018; Mikolajczak et al., 2018, 2020; Van Bakel et al., 2018; Brianda et al., 2020). Potential antecedents of parental burnout have been traced in socio-demographic factors, particularities of the child, parental traits and behaviors, and family functioning (Mikolajczak et al., 2018, 2020). However, little is known about the role played by disruptive events, such as a pandemic. In a longitudinal study of Portuguese parents before and during the pandemic Aguiar et al. (2021) found that the prevalence of parental burnout was indeed higher during the pandemic. In the current study, we examine Italian parents in two subsequent years, 2021 and 2022. Italy was one of the first countries that was severely hit by the Covid-19 pandemic in the spring of 2020, and to date counts over 20 million confirmed cases and about 170.000 confirmed casualties, which is one of the highest death rates in Europe (WHO, 2022). We collected data during a new steep rise in cases in March and April 2021, and a plateauing level of cases in March and April 2022. Italy during these months was in a formal state of emergency in 2021 as well as 2022. Measures in the spring of 2021 were very strict (schools were closed, leaving one’s house only allowed with authorization) and still strict in the spring of 2022, although at the end of March 2022 restriction measures were gradually loosened and more mobility was allowed for the vaccinated and when waring mouth-nose-masks (Mazzuca, 2022). Protests against compulsory vaccination and restriction measures in Italian society increased during this timeframe, resulting in a substantial polarization in Italian society (Bondielli et al., 2022; Spitale et al., 2022). This polarization has left no family untouched. Hence, examining the extent to which Italian parents over the course of two consecutive years experienced the Covid-19 pandemic and whether this affects their ability to perform in their role as parent will give a unique insight.

The extent to which the pandemic affects work–family conflict and subsequent parental burnout may depend on how organizations respond to their staff’s needs for security, reassurance, stability and affiliation (Emmet et al., 2020). Perceived organizational support (POS; Rhoades and Eisenberger, 2002) constitutes a potential resource that has been found to attenuate job burnout risk (Walters and Raybould, 2007; Cheng and O-Yang, 2018), and to moderate the relationship between role conflict and job burnout (Wu et al., 2018), especially the dimension emotional exhaustion (Jawahar et al., 2007). As the present research focuses on parents, organizational support will be identified not only as positive organizational policies and attitudes aimed at valuing employees’ work, goals and wellbeing but also as specific behaviors and philosophies focused on facilitating effective parenting during the pandemic. Examples of family supportive practices and philosophies could be: allowing time off to attend to family needs, accepting boundary blurriness and considering flexible time arrangements. Overall, this study aims to answer the following research questions:

RQ1: To what extent is parental burnout influenced by how intense the Covid-19 crisis was experienced and is this relationship mediated by work–family conflict in Italy in 2020 and 2021?

RQ2: To what extent do family supportive organizational perceptions buffer the relationship between work–family conflict and parental burnout in Italy in 2020 and 2021?

Altogether, the purpose of the present study is to contribute to the parental burnout literature, enhancing knowledge around its potential risk factors. In particular, it aims at providing insight into the extent to which parental burnout insurgence may be influenced by the perceived intensity of the Covid-19 situation, in terms of social isolation and work-related psychological risk factors. Furthermore, during the course of the pandemic, vaccinations and vaccination passports led to less strict restriction measures for vaccinated or recovered individuals, but less for the unvaccinated. Heated debates in society sometimes trickled down into families, creating tensions between parents, or parents and other relatives, increasing the burden on parents and thus there susceptibility to parental burnout. As this pandemic is likely to continue (Charumilind et al., 2020), and the incidence of future pandemics is not to be excluded (Gill, 2020), it is of interest to have a full understanding of the psychological impact of lockdowns, quarantines, restrictions and infection precaution measures. Specifically, parental burnout is a social issue that not only shows negative symptoms for parents, both in terms of health and productivity, but it also affects the relationship with their partner and the wellbeing and safety of their children (Mikolajczak et al., 2019). For this reason, knowing the risk factors that may facilitate its incidence may be of great societal relevance. Moreover, comprehending the mitigating effect of organizational support on parental burnout is significant for HR management, which can play a key role in guiding organizations in preventing this phenomenon from happening by applying policies and practices of support.

## Theoretical framework

### Parental burnout

In 2014, Bianchi and colleagues argued that burnout is not solely a work-related condition (Bianchi et al., 2014). They suggested burnout can be developed in any domain as long as frequent and intense stress is elicited. Although parenting is knowingly considered as a complex and demanding activity, subjected to various intense stressors, the concept of parental burnout is quite new in the literature. It was identified as a unique specific syndrome only in 2017 (Roskam et al., 2017), described as a state of intense exhaustion, decreased self-efficacy and diminished involvement in the relationship with one’s children, originated by a strong imbalance between parental demands and the resources available to meet them (Hubert and Aujoulat, 2018; Roskam et al., 2018; Mikolajczak et al., 2020). Parental burnout has been found to associate with various behaviors such as depressive symptoms, sleep disorders, addictive behaviors, conflicts with the partner, escape ideation and child neglect or abuse (Kawamoto et al., 2018; Mikolajczak et al., 2018, 2020; Van Bakel et al., 2018).

Originally, the construct of parental burnout was derived from the tridimensional structure of classical job burnout, defined by exhaustion, depersonalization, and professional efficacy (Maslach et al., 2001). The first dimension, emotional exhaustion, implies feelings of weariness and depletion connected to the care of one’s children. The second dimension refers to emotional distancing from one’s children, which describes a situation where parents detach emotionally from their children, though still providing practical care. The third dimension, personal accomplishment, entails feelings of inefficacy and inadequacy in the parental role (Roskam et al., 2017). However, when Roskam and colleagues reconstructed the concept of parental burnout through an inductive approach, they found evidence of a fourth dimension: the contrast with previous self. In fact, the current state of a person must markedly diverge from the previous state in order for the individual to experience burnout (Roskam et al., 2018). Mikolajczak et al. (2020) recently showed that parental burnout can be distinguished from job burnout both in its underlying dimensions that are specific for the sphere of life from which they originate, but also in terms of their consequences as “parental burnout has a unique impact on parenting (parental satisfaction, parental neglect and violence), and job burnout has a unique impact at work (job satisfaction, turnover intention)” (p. 685).

### Perceived Covid-19-crisis intensity

One event that can exacerbate parental stress to the point where a burnout may manifest is the Covid-19 pandemic. Mason (1968) suggests that elements and events characterized by novelty, unpredictability, threat and lack of control trigger a stress response in the individual. The outbreak of Covid-19 represents a stressor that shows all these attributes: it brings an unprecedented situation, with unpredictable outcomes; it is perceived as threatening on social, financial and health-related aspects and its yet unclear mechanisms of diffusion challenge every sense of control (Pahayahay and Khalili-Mahani, 2020). The prolonged exposure to such stressors may lead to the state of burnout (Maslach and Leiter, 2016). The perception of Covid-19 intensity is here defined in terms of psychological stressors during work and feelings of loneliness caused by a state of protracted isolation.

The Covid-19 pandemic not only may have caused a sudden rise in psychological stressors, it also created an enormous change in the organization of work. Before the pandemic, only a small proportion of the workforce was working remotely, and working from home was often only for a part of the actual work time (International Labour Office, 2020). However, especially for knowledge workers, the Covid-19 pandemic has forced a quick shift to full-time working from home (Carnevale and Hatak, 2020). Research reports that working from home often leads to feelings of confusion and unclarity around how to organize and prioritize one’s tasks, how to use technological tools, whom to ask for support, how to approach colleagues and how to deal with new tasks (Bick et al., 2020; International Labour Office, 2020). According to role theory (Roy et al., 1965), being constantly exposed to incongruent or vague expectations leads to role conflict and role ambiguity, conditions that may easily result in stress and anxiety (House and Rizzo, 1972). Indeed, relationships were found between role conflict and psychological strain, job burnout and single dimensions of job burnout (Jawahar et al., 2007). Furthermore, working remotely generally requires the sustained use of technological devices. These elements have been shown to induce technostress, which entails negative psychological states (Kolakowski, 2020; Molino et al., 2020; Spagnoli et al., 2020).

Other psychological effects of working in confinement are from the employee isolation literature. Employee isolation is a psychological construct that refers to employees’ perception of lacking opportunities for professional, social and emotional exchange with their co-workers (Mulki and Jaramillo, 2011; Brooks et al., 2020). In virtual work environments, employees often fail to conform to the organizational culture, perceiving themselves as a single entity (Jaiswal and Arun, 2020). Employees may fear that their career opportunities will be limited and they miss the informal discussions and face-to-face interaction that facilitate not only information sharing, but also the emergence of positive feelings of trust and belonging (Cooper and Kurland, 2002; Gajendran and Harrison, 2007; Fosslien and West-Duffy, 2019).

In addition to work-related feelings of isolation, employees may experience a general sense of loneliness, connected to further governmental regulations put in place to limit the transmission of Covid-19, which implied restriction of mobility, interruption of social and leisure activities, separation from loved ones, loss of freedom, lack of information and social support (Banerjee and Rai, 2020; Saladino et al., 2020). Loneliness, generally a consequence of social isolation, is considered to be one of the major risk factors for various disorders, such as anxiety, depression, chronic stress, insomnia and even late-life dementia (Wilson et al., 2007; Brooks et al., 2020).

Stickley and Koyanagi (2016) argued that long periods of isolation in custodial care or quarantine have detrimental effects on mental wellbeing. Literature concerning the epidemic of SARS (severe acute respiratory syndrome) reported that quarantined subjects, compared to non-quarantined, were more likely to develop symptoms of exhaustion, detachment from others, anxiety, irritability, insomnia, poor concentration and indecisiveness, deteriorating work performance, reluctance to work and consideration of resignation (Bai et al., 2004). Other psychological symptoms showed by isolated subjects were emotional disturbance, depression, stress, low mood, irritability, insomnia and post-traumatic stress symptoms (DiGiovanni et al., 2004; Hawryluck et al., 2004; Tam et al., 2004; Lee et al., 2005; Liu et al., 2012; Brooks et al., 2020).

Findings show that parents represent a particularly vulnerable category in times of crisis. Taylor et al. (2008) report prevalence of very high psychological distress for respondents with one child during the outbreak of equine influenza in Australia. Data collected among parents who had experienced quarantine during the pandemic of H1N1 or SARS, showed that the subjects presented high levels of Post-Traumatic Stress Disorders (Sprang and Silman, 2013). Research conducted during the diffusion of Covid-19 confirms these discoveries. A study assessing the psychological impact of the Covid-19 pandemic on Italian parents reported that 17% of the respondents experienced significant parenting-related exhaustion (Marchetti et al., 2020). Lockdown measures were found to predict the peak in parents’ levels of depression and anxiety (Johnson et al., 2020). The comparison between subjects with and without children showed that the Covid-19 crisis led to a greater decrease in the wellbeing of individuals with children, especially younger ones (Huebener et al., 2021).

As far as parental burnout is concerned, only few studies exist. Prikhidko and Long (2020) demonstrated that a moderate relationship exists between the concern related to Covid-19 and parental burnout. Furthermore, Aguiar et al. (2021) found that the prevalence of parental burnout was higher during the pandemic. In a study among Italian parents, Cusinato et al. (2020) found that lockdown measures and changes in daily routine negatively affected parents’ psychological dimensions. To conclude, the foregoing leads to the following hypotheses:

*H1*: Perceived Covid-19-Crisis Intensity is positively related to parental burnout

Furthermore, we expect that societal debates and the polarization in Italian society (Bondielli et al., 2022; Spitale et al., 2022) may trickle down and create tensions in families. We argue that diverging attitudes concerning Covid-19 vaccination and vaccination passports may create additional psychological stressors for parents. To our knowledge there is no study to date examining whether tensions related to vaccination and infection precaution measures affect parents in such a way that it increases parental burnout. From research among couples we can infer that this may be the case. Schokkenbroek et al. (2021) found that couples in Belgium became more aware of diverging attitudes during the Covid-19 pandemic, resulting in feeling less connected and more stressed. Hence, we argue that:

*H1a*: Family tensions related to diverging attitudes on vaccination and infection precaution measures (VIPM) positively relate to parental burnout.

### The mediating role of work–family conflict

Perceived Covid-19-Crisis Intensity (PCCI) and Family tensions related to diverging attitudes concerning infection and precaution measures (VIPM) may not only directly elicit parental burnout through anxiety and psychological distress as a response to the novelty, unpredictability, threat and lack of control the pandemic brings along, but also indirectly, as both PCCI and VIPM may evoke increased role conflict between parents work and family demands. For most knowledge workers, the Covid-19 pandemic meant an abrupt change to (mandatory) full-time remote work, (Carnevale and Hatak, 2020; Ghislieri et al., 2021), for those workers whose work could not be transformed to remote working it often meant substantial -and stressful - health precaution measures at work. In many instances they quickly needed to adapt to new circumstances at work, while at the same time, they had to devote more time and energy to their children, due to the closure of schools and centers for childcare (Carnevale and Hatak, 2020). Parents thus often had to manage increased demands in the work and family domain simultaneously. From role theory it can be argued that participating in multiple roles may lead to inter-role conflict, as it becomes harder to fulfil multiple roles successfully due to competing demands or discordant behaviors among roles (Roy et al., 1965). Work–family conflict is a particular type of inter-role conflict that originates from simultaneous incompatible demands stemming from both the family and the work domain, leading to compromised effectiveness in either one or both roles (Greenhaus and Beutell, 1985). Specifically, conflict might occur when the amount of time and energy devoted to one role is limited due to the high demands associated with the other role (time-based and energy-based conflict), when stress arising in one role is transferred to the other role, this causes strain symptoms (strain-based), and/or when behaviors that are effective in one role are inappropriately enacted in the other role (behavior-based; Greenhaus and Beutell, 1985; Greenhaus et al., 2006). Particularly when boundaries between domains are blurred, work–family conflict is more likely to occur (Hunter et al., 2019). The Covid-19 pandemic made the boundaries of the domains of work and family blur in many ways, hence making work–family conflict more likely.

Subsequently, work–family conflict can be seen as a source of stress that induces burnout. The Conservation of Resource (COR) Theory offers a framework for explaining work–family conflict as a source of stress (Grandey and Cropanzano, 1999). This stress model is based on the assumption that people strive to maintain, protect, and create resources. The threat of losing these resources, their actual loss, or the null gain of resources after a positive investment are factors that lead to stress (Hobfoll, 1988). According to the COR model, inter-role conflict causes experiences of stress because resources are lost in the process of finding a balance between work and family (Grandey and Cropanzano, 1999). Assuming that one’s time and energy are limited resources, individuals who engage in multiple roles will invest resources in one role, thus unavoidably experiencing a resource drain in the other role (Edwards and Rothbard, 2000). Hence, employees who are confronted with higher work-related demands will experience a greater loss in the family domain and vice versa (Bakker and Geurts, 2004; Butler et al., 2005). These losses are the root of negative feelings or states such as dissatisfaction, depression, anxiety, physiological tension and burnout (Hobfoll and Shirom, 1993).

Considerable evidence exists in the literature for a positive relationship between work–family conflict and negative health-related outcomes. In fact, work-family-conflict was consistently found to correlate with depression, anxiety and psychological strain (Grandey and Cropanzano, 1999; Frone et al., 2020). It also appears to correlate with physical conditions reflecting the sympathetic nervous system’s reactions to stress (e.g., blood pressure, cholesterol levels, hypertension) and with self-reported negative health symptoms and unhealthy behaviors (Thomas and Ganster, 1995; Spector et al., 2004).

Furthermore, conflicts between the work and family domains have been found to mediate the relationship between demanding work characteristics and indicators of psychological wellbeing and job burnout (Geurts et al., 1999; Janssen et al., 2004; Rupert et al., 2009) as well as mediating the relationship between demanding family characteristics and indicators of psychological wellbeing (Asiedu et al., 2018) particularly during the pandemic (Swit and Breen, 2022). As the Covid-19 pandemic has characteristics of both demands, we expect that:

*H2*: Work-family conflict mediates the relationship between PCCI and parental burnout

Furthermore, tensions related to diverging attitudes in families concerning vaccination and precaution measures are likely to affect the support parents may experience from their spouse in juggling work and family demands negatively. As spousal support is one of the most important resources for parents, such diminished spousal support is likely to exacerbate work–family conflict and consequently may lead to a higher likelihood of parental burnout. From meta-analytic research examining the antecedents of work–family conflict spousal support is indeed seen as an important resource to prevent work–family conflict (Michel et al., 2011). To clarify the matter further, research among Dutch parents during the Covid-19 pandemic shows that if parents had more disagreements about any of five issues (working from home, working on location, care for the children, free time, household tasks) they perceived more stress (André and van der Zwan, 2021). This suggests that if parents have disagreements about working from home or on location, or about whether to take precaution measures (such as vaccination) within their own nuclear family (with their spouse) or with their extended family (parents, in-laws) this has a great potential to create tensions and thus impact work–family conflict from the family side of the balance.

Hence, we expect that:

*H2a*: Work-family conflict mediates the relationship between family tensions towards VIPM and parental burnout

### The moderating role of family supportive organizational practices during the pandemic

As this unprecedented crisis has brought alterations and instability in various aspects of work and everyday life, support from the organization is essential to help staff go through this transition process and adjust to the “new normal” (Gigauri, 2020). Organizational support is shown to be extremely protective of employees’ mental health during the outbreak of infectious diseases (Brooks et al., 2018). Some of the recommended initiatives to favor employees’ wellbeing and stability in times of crisis consists of the adoption of flexible schedules, less strict policies concerning performance management, training and support to virtual working skills, together with consistent, transparent and empathetic communication (Adams, 2020; Fallon, 2020; Howlett, 2020). Further solutions could include stress-mitigating offerings such as webinars on resilience, tutorials on mindfulness (De Cieri et al., 2019), employee assistance programs, and virtual counseling services (Caligiuri et al., 2020).

The Organizational Support Theory proposes that, over time, based on the multiple interactions with supervisors and employers and on the quality of working conditions and HR offerings, employees tend to form a generalized and stable perception of organizational support (Rhoades and Eisenberger, 2002; Kurtessis et al., 2015). Perceived organizational support is thus defined as the pattern of employee’s beliefs concerning the extent to which the organization values their contributions and cares about their wellbeing (Eisenberger et al., 1986). When employees feel they are being favored, well-treated and valued by their organization, their socio-emotional needs of belongingness and esteem are fulfilled (Poldma, 2016), and they experience a greater wellbeing (Kurtessis et al., 2015). Perceived organizational support constitutes a potential resource that has been found to attenuate job burnout risk (Walters and Raybould, 2007; Cheng and O-Yang, 2018; Zeng et al., 2020) and to moderate the relationship between inter-role conflict and job burnout (Wu et al., 2018), especially the dimension of emotional exhaustion (Jawahar et al., 2007).

As the present research focuses on parents, organizational support will be identified as positive organizational policies and attitudes aimed at valuing employees’ work, goals and wellbeing with a focus on facilitating effective parenting. Allen (2001) suggests that family-friendly benefits may not have the intended impact if they are not accompanied by family-friendly organizational values. Therefore, together with organizational policies, family supportive organizational perceptions will be taken into consideration. This dimension is defined by Thompson et al. (1999) as the “the shared assumptions, beliefs, and values regarding the extent to which an organization supports and values the integration of employees’ work and family lives” (p. 392). Examples of family supportive perceptions could be: allowing time off to attend to family needs, accepting boundary blurriness, considering flexible time arrangements as a strategic solution. In addition, the extent to which parents perceive the organization to offers support and guidance during the Covid-19 pandemic might be particularly important (e.g., Carnevale and Hatak, 2020; Fontanesi et al., 2020). This study will examine the extent to which parents family supportive organizational perceptions during the pandemic will buffer the risk of parental burnout that stems from work–family conflict:

*H3*: Family supportive organizational practices during the pandemic (FSOP-p) have a moderating effect on the relationship between work-family conflict and parental burnout

**Figure 1:**
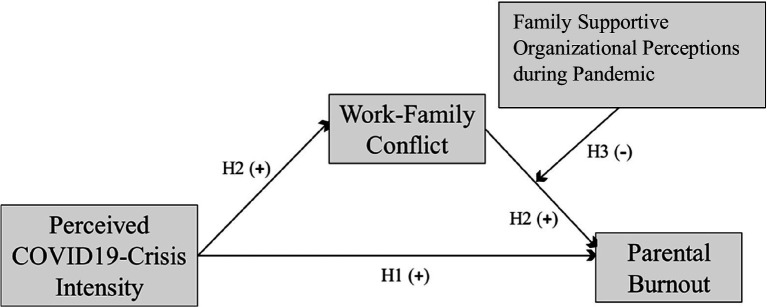
Hypothesized Relationships among Variables.

The relationships between Perceived Covid-19-Crisis Intensity, parental burnout, work–family conflict and family supportive organizational perceptions as described above are shown in the conceptual model below (Figure 1).

## Materials and methods

### Research design

A quantitative research was conducted to investigate the aforementioned hypotheses by data collection *via* online questionnaires, which were considered as the fastest and cheapest way to reach as many respondents as possible, especially during times of Covid-19 restriction measures. The data collection took place during March/April 2021 and March/April 2022. In March/April 2021 and 2022 Italy was in a formal state of emergency. In 2021 there was a strict lockdown, with schools closed, mandatory home-working, and the population was not allowed to leave the house without formal authorization (see https://www.governo.it/it/coronavirus-misure-del-governo). In 2022 there were still restrictions, such as compulsory mouth-nose masks and vaccination passports in public spaces, public transport and so forth. From April 1st 2022 onwards there was a gradual loosening of restriction measures (week by week, depending on the color code of the region; Mazzuca, 2022). Although our aim was to conduct a longitudinal study, of the 286 respondents participating in March/April 2021 only 35 respondents filled out the questionnaire during March/April 2022, hence we proceeded with a stacked cross-sectional design using two data waves one year apart.

### Procedure

Ethical approval was received before starting recruiting participants. The questionnaire was distributed during March/April 2021 through a link to a web-based questionnaire in Italian, which was sent *via* email and other social media to people in the researchers’ networks. In March/April 2022 we reached out to the same pool of respondents, through email and social media with a small flyer visualizing the 2021 main findings and a link to the follow-up questionnaire. The aim of the study was explained before the questionnaire started both in the participation invitation and, more extensively, on the front page of the web-based questionnaire. On this page, anonymity and confidentiality were guaranteed. In addition, a verification and informed consent form was included and filled in by respondents in order to verify themselves as existing persons.

### Participants

Of the 286 respondents that started the survey in 2021, 222 filled in the complete questionnaire and met the inclusion criteria (working parent with a child living at home that was 18 years or younger). As it was assumed that pandemic-related modifications in the working conditions would not have a sufficient impact on subjects working less than 12 h per week, also participants who worked less than 12 contract or actual hours per week were excluded. Hence, the analyses for Time 1 were carried out on a sample of 222 subjects. The Time 1 sample consists of 50 men (21%), 186 women (78.5%) and one with non-specified gender (0.4%). Their average age was 43.9, ranging from 30 to 60 years old (*SD* = 6.24). 219 (92.4%) of the respondents were married or cohabiting, 16 (6.8%) of the respondents were single, divorced or widowed and one (0.4%) was in a relationship but not cohabiting. The number of children living at home ranged from 1 to 4 (*M* = 1.81, *SD* = 0.73), the average age of the youngest child was 7.66 years old (*SD* = 5.00), ranging between 0 and 18. 84 (37.3%) stated that in the past year they have dedicated less or the same amount of time to their children’s care as the years before, while the rest (62.7%) spent more or much more time taking care of their children. Furthermore, the number of working hours per week was on average 36.17 (*SD* = 9.66). 142 respondents (60.7%) worked from home at least one or two days per week.

In the second data collection, 127 participants started the survey; however, only 83 filled in all scales and met the inclusion criteria. The Time 2 sample consisted of 15 men (18.1%), 68 women (81.9%). Their average age was 43.9, ranging from 26 to 60 years old (*SD* = 8.15). 75 (90.4%) of the respondents were married or cohabiting, 6 (7.2%) of the respondents were single, divorced or widowed and 2 (2.4%) were in a relationship but not cohabiting. The number of children living at home ranged from 1 to 4 (*M* = 1.70, *SD* = 0.73), the average age of the youngest child was 7.84 years old (*SD* = 5.71), ranging between 0 and 18. 67 respondents (80.7%) stated that in the past year they had dedicated less or the same amount of time to their children’s care as the years before, while 16 (19.3%) spent more or much more time taking care of their children. Furthermore, the number of working hours per week was on average 35.14 (*SD* = 11.94). 12 respondents (15%) worked from home at least 1 day a week. In general our samples seem to be quite consistent over time, although our response is lower at the second timepoint. The largest changes are that most people returned to office at our second timepoint, and, compared to the first covid-year, parents in the second covid-year did not spent much more time on their children.

### Measures

*Perceived Covid-19-Crisis Intensity (PCCI) at Time 1* was measured with a scale composed of 18 items derived from two scales that were adapted for the current Covid-19-Crisis. The first part consisted of a shortened and adapted version (15 items) of the Short Inventory to Monitor Psychological Hazards (SIMPH; Notelaers et al., 2007). Items were modified in order to specifically address the comparison between pre- and post-Covid-19 outbreak. Example items were “Compared to the period preceding Covid-19, I now have to work harder to complete any work task” or “Compared to the period preceding Covid-19, it’s now easier to ask my colleagues for help” (reversed). Respondents were asked to answer with regard to the year following the initial outburst of the pandemic. All items were rated on a 5-point Likert scale, where 1 = strongly disagree and 5 = strongly agree. In order to test the validity of the scale and to examine its underlying structure, an exploratory factor analysis was conducted. Three components were found and were named: (1) *Struggle and Confusion*, (2) *Lack of Social Support*, and (3) *Lack of Autonomy*. Reliability of the overall scale was tested through Cronbach’s Alpha, which was 0.81, indicating good internal consistency (George and Mallery, 2018). For the subscales, Cronbach’s Alpha was, respectively, 0.83 (Struggle and Confusion); 0.83 (Lack of Social Support); and 0.74 (Lack of Autonomy).

*Perceived Covid-19-Crisis Intensity (PCCI) at Time 2*. The same items were used at Time 2, but the instructions were adapted to the current situation. An example item is “Compared to last year, I now have to work harder to complete any work task” or “Compared to last year, it’s now easier to ask my colleagues for help.” The overall Cronbach’s Alpha at Time 2 was 0.80, and for the subscales they were, respectively 0.87 (Struggle and Confusion); 0.79 (Lack of Social Support); and 0.74 (Lack of Autonomy).

*Parental burnout (PB)*, was evaluated using a scale constructed through the combination of Parental Burnout Inventory (PBI; Roskam et al., 2017) and Parental Burnout Assessment (PBA; Roskam et al., 2018). PBI contains three dimensions (*emotional exhaustion*, *emotional distancing*, and *inefficacy*), while PBA also entails an additional dimension (*contrast with previous self*). Hence, the scale consisted of 12 items, nine of which pertained to PBI (for instance: “I accomplish many valuable things as a parent”), while three were drawn from PBA (for example: “I tell myself that I’m no longer the parent I used to be”). Respondents were asked to respond on a five-point Likert scale ranging from 1 = strongly disagree to 5 = strongly agree. Cronbach’s alpha was 0.92 at Time 1 and 0.90 at Time 2, which means the scale has good internal consistency.

To measure *Work–family conflict (WFC)*, six items were adopted out of the 18-item-scale developed by Carlson et al. (2000). The items assessed to what extent private life and family activities affect the work domain and vice versa. One example item is: “The time I spend with my family often causes me not to spend time on activities at work that could be helpful to my career.” As most parents had to work from home during the months of April/March 2021 and 2022, we slightly modified the wording of the items that suggested work and family domains were at different locations. For instance: “I am often so emotionally drained *when I get home* from work that it prevents me from contributing to my family” was changed into: “I am often so emotionally drained *when I finish working* that it prevents me from contributing to my family.” Respondents were asked to answer on a five-point Likert scale ranging from 1 = strongly disagree to 5 = strongly agree. Cronbach’s alpha was 0.83 at Time 1 and 0.78 at Time 2, indicating the scale to be reliable.

*Family Supportive Organizational Perceptions during the pandemic* was assessed through an adaptation of the 7-item scale derived from Allen’s (2001) Family-Supportive Organizational Perceptions (FSOP) scale. Example items are: “My organization assumes that the most productive employees are those who put their work before their family life” (reversed) and “My organization believes that employees should be given ample opportunity to perform both their job and their personal responsibilities as well.” Two more items were added in order to focus on the pandemic-related behaviors: “During the pandemic, my organization has provided specific instructions on how to act and behave” and “During the pandemic, my organization offered to help me more than usual.” For all items, respondents were asked to assess on a Likert five-point scale ranging from 1 = strongly disagree to 5 = strongly agree. At Time 1 Cronbach’s alpha was 0.88 and at Time 2 0.83.

Finally, control variables were included to see whether there are spurious relations affecting the relationships in the proposed conceptual model. The control variables were gender, age, number of children living at home, youngest child’s age, change in time spent with children, work hours and telework.

### Statistical analysis

We performed linear regression analysis to test hypothesis 1 and tested the mediation (hypothesis 2) and moderation (hypothesis 3) models using Hayes’ PROCESS (Hayes, 2017). We used model 4 for mediation and model 14 for moderation.

## Results

Table 1 shows the means (*M*), standard deviations (*SD*) and significant correlations for each main variable and control variables for Time-1 (below the diagonal) and Time-2 (above the diagonal). PCCI is highly and significantly correlated with parental burnout and work–family conflict at Time-1 as well as Time-2. Furthermore, Perceived Organizational Support was negatively related to PCCI, parental burnout and work–family conflict. Tensions related to vaccination and infection precautions (VIPM) were, contrary to our expectations, not correlated with any of the other measures.

**Table 1:** Correlation matrix. Below the diagonal the correlations for Time-1 (*N* = 222), above the diagonal the correlations for Time-2 (*N* = 83).

	Variable	M /% (T1)	SD (T1)	M/% (T2)	SD (T2)	Diff	1	2	3	4	5	6	7	8	9	10	11	12	13
1	PCCI	3.10	0.61	2.93	0.64	*	1	0.39 ***	0.50 ***	−0.65 ***	0.18	0.04	−0.233 *	−0.07	0.03	−0.11	0.10	−0.10	−0.03
2	PB	2.17	0.81	2.20	0.75	n.s.	0.47 ***	1	0.53 ***	−0.385 ***	0.21 *	0.04	−0.010	−0.01	0.04	−0.09	−0.01	−0.08	0.17
3	WFC	2.70	0.88	2.72	0.87	n.s.	0.37 ***	0.52 ***	1	−0.459 ***	0.14	−0.00	−0.16	−0.25 *	−0.05	−0.01	0.02	−0.09	−0.04
4	FSOP-p	3.19	0.88	3.09	1.09	n.s.	−0.34 ***	−0.27 ***	−0.278 ***	1	−0.23 *	−0.04	0.25 *	0.19	−0.12	0.14	−0.15	0.23 *	0.14
5	Gender (female)	78%		81%		n/a	0.19 **	0.20 **	0.094	−0.125	1	−0.13	−0.08	0.07	−0.01	−0.08	−0.16	−0.21	0.06
6	Age	43.61	6.15	43.30	8.16	n.s.	0.09	0.04	−0.177 **	0.045	−0.02	1	0.02	0.26 *	0.84 ***	−0.21	0.20	0.01	−0.12
7	Education	4.43	1.03	4.43	0.99	n.s.	−0.07	0.02	0.183 **	0.015	−0.10	−0.08	1	0.07	−0.11	0.08	0.29 **	0.29 **	−0.03
8	Number of children	1.82	0.71	1.70	0.73	n.s.	0.14 *	0.14 *	−0.054	0.093	0.09	0.14 *	−0.18 **	1	0.15	−0.16	−0.08	−0.13	−0.02
9	Age youngest child	7.50	5.00	7.84	5.71	n.s.	0.06	−0.00	−0.229 ***	0.019	0.08	0.744 ***	−0.19 **	0.10	1	−0.32 **	0.16	−0.14	−0.08
10	Time spent on Number of children	3.80	0.86	2.98	0.92	***	0.01	0.11	0.167 *	−0.025	0.15 *	−0.15 *	0.10	0.14 *	−0.22 ***	1	−0.23 *	0.20	0.10
11	Work hours	36.09	9.72	35.14	11.94	n.s.	−0.21 ***	0.01	0.156 *	−0.009	−0.29 ***	−0.13	0.13 *	−0.11	−0.16 *	−0.03	1	0.20	−0.12
12	Telework (almost always)	49%		15%			−0.11	0.05	0.132 *	0.145 *	−0.02	0.12	0.31 ***	−0.00	−0.05	0.17*	0.12	1	0.11
13	VIPM (only at T2)																		1

All scales measured on 5-point Likert scale. For Time-1 telework hours was calculated based on the number of days a week that is spend on telework, at Time-2 the number of hours was directly used. Gender (0 = men, 1 = women), Age and Education were measured in intervals with increasing age and education.

*** *p* < 0.001;

** *p* < 0.001;

* *p* < 0.05.

We test the first hypothesis with linear regression analysis (see Table 2). Model 1 shows that the direct effect of PCCI on Parental Burnout in 2021 is 0.610 (*p* < 0.001), which means that a higher perceived covid crisis intensity is related with a higher score on the parental burnout scale. Model 2 shows that in 2022 the direct effect of PCCI on Parental Burnout is still positive and significant in a linear regression analysis (0.415, *p* < 0.01), controlled for gender, age, number of children, age youngest child, work hours, telework hours and time for children compared to last year. Although the size of the effect is smaller, since we are working with two different samples we should be careful in interpreting the size of the effect. These results confirm our first hypothesis that Perceived Covid-19 Crisis Intensity (PCCI) is positively related to parental burnout.

**Table 2:** Regression analysis on parental burnout (Y).

	Model 1 (Time-1)	Model 2 (Time-2)	Model 3 (Time-1)	Model 4 (Time-2)	Model 5 (Time-2)
	B(SD)	B(SD)	B(SD)	B(SD)	B(SD)
Constant	−1.163 (0.562)*	0.739 (0.855)	−1.249 (0.521)*	0.613 (0.776)	0.287 (0.780)
PCCI	0.610 (0.080)***	0.415 (0.128)**	0.389 (0.083)***	0.160 (0.132)	0.157 (0.129)
Gender	0.334 (0.125)*	0.294 (0.221)	0.252 (0.117)*	0.207 (0.201)	0.188 (0.198)
Age	0.007 (0.012)	0.006 (0.020)	0.011 (0.011)	−0.008 (0.019)	−0.004 (0.018)
Number of children	0.066 (0.068)	−0.015 (0.119)	0.108 (0.064)	0.121 (0.113)	0.116 (0.111)
Age youngest child	−0.009 (0.005)	−0.004 (0.029)	0.000 (0.014)	0.016 (0.026)	0.011 (0.026)
Work hours	0.014 (0.005)	−0.003 (0.007)	0.007 (0.004)	−0.002 (0.007)	−0.001 (0.007)
Telework hours	0.030 (0.031)	0.000 (0.007)	0.005 (0.029)	0.003 (0.007)	0.001 (0.007)
Time for children compared to last year	0.061 (0.059)	−0.040 (0.097)	0.025 (0.055)	−0.037 (0.088)	−0.046 (0.086)
Work–Family Conflict (WFC)			0.356 (0.059)***	0.417 (0.101)***	0.421 (0.099)***
Vaccination and Infection Precaution Measures (VIPM)					0.127 (0.065)^
*N*	*222*	*83*	*222*	*83*	*83*
*R2*	*0.293*	*0.177*	*0.396*	*0.332*	*0.366*

*** *p* < 0.001;

** *p* < 0.001;

* *p* < 0.05;

^ *p* < 0.10.

Models 3 and 4 show that Work–Family Conflict (WFC) has a positive direct effect on Parental Burnout at Time-1 (*b* = 0.356, *p* < 0.001) and Time-2 (*b* = 0.417, *p* < 0.001). We will test the mediation effect with Hayes in the next paragraph. Model 5 is only present for Time-2, where we investigate if tensions in the family about getting vaccinated or infection precaution measures affects parental burnout. We find the effect is borderline significant (*p* = 0.056). This gives a first indication that we might not have to reject our hypothesis 1a that family tensions related to attitudes toward vaccination and infection precaution matters are positively related to parental burnout.

**Figure 2:**
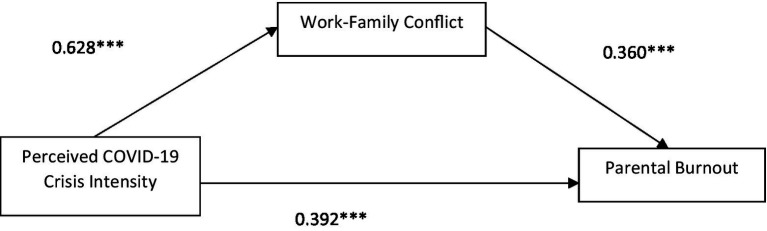
Mediation model (T1, 2021).

**Figure 3:**
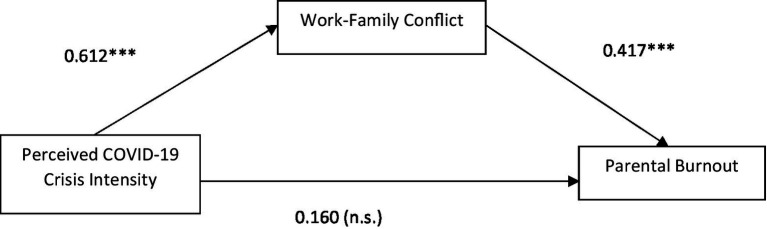
Mediation model (T2, 2022).

Hypothesis 2 in which we predict that work–family conflict mediates the relationship between PCCI and parental burnout has been tested with Hayes Process model 4. The results for 2021 are presented in Figure 2 and for 2022 in Figure 3. In 2021, we find a significant direct and indirect relationship between PCCI and parental burnout. Which means that the relationship is only partly mediated by Work–Family Conflict. The direct effect was 0.610 and is now 0.392. In 2022 this relationship is fully mediated by the level of work–family conflict of the respondent as can be seen in Figure 3 below. The relationship between PCCI and work–family conflict is 0.59 and the relationship between work–family conflict and parental burnout is 0.40. See for all coefficients Table 3. This allows confirmation of hypothesis 2.

**Table 3:** Hayes mediation analysis on parental burnout (Y) with Work–Family Conflict (M) and PCCI (X; models 1 and 2).

	Time-1 (2021)		Time-2 (2022)	
	Work–Family Conflict	Parental Burnout	Work–Family Conflict	Parental Burnout
	B (SD)	B(SD)	B (SD)	B(SD)
Constant	−0.348 (0.604)	−1.172 (0.519)*	0.096 (0.099)	0.405 (0.863)
PCCI	0.628 (0.087)***	0.392 (0.083)***	0.612 (0.134)***	0.160 (0.132)
Gender	0.190 (0.135)	0.240 (0.116)*	0.208 (0.230)	0.207 (0.201)
Age	−0.001 (0.013)	0.011 (0.011)	0.035 (0.021)	−0.008 (0.019)
Number of children	−0.116 (0.07)	0.111 (0.064)	−0.326 (0.124)*	0.122 (0.113)
Age youngest child	−0.028 (0.016)	0.000 (0.014)	−0.047 (0.030)	0.016 (0.026)
Work hours	0.146 (0.036)***	0.046 (0.032)	−0.002 (0.008)	−0.002 (0.007)
Telework hours	0.106 (0.107)	0.025 (0.092)	−0.007 (0.008)	0.003 (0.007)
Time for children compared to last year	0.120 (0.063)	0.025 (0.054)	−0.008 (0.100)	−0.037 (0.088)
Work–Family Conflict (WFC)		0.360 (0.058)***		0.417 (0.101)***
VIPM				
				
*N*	*222*	*222*	*83*	*83*
*R2*	*0.298*	*0.394*	*0.332*	*0.332*

*** *p* < 0.001;

** *p* < 0.001;

* *p* < 0.05;

^ *p* < 0.10.

We also tested the three subscales of PCCI in this mediation model: struggle and confusion, lack of autonomy and lack of social support in Tables 4a,b. The subscale struggle and confusion had the largest explanatory power and seems to drive the relationship between PCCI and parental burnout. This is the same in 2021 and 2022. The other two subscales did not have a significant relationship with parental burnout. We also tested the curvilinear effect of the ‘lack of autonomy’ scale, we did not find a curvilinear effect on either timepoint.

**Table 4a:** Hayes mediation analysis on parental burnout (Y) with Work–Family Conflict (M) and PCCI (X; including all controls from the past Table, results not shown) at Time-1 (2021).

	Time-1 (2021)					
	Work–Family Conflict	Parental Burnout	Work–Family Conflict	Parental Burnout	Work–Family Conflict	Parental Burnout
	B(SD)	B(SD)	B(SD)	B(SD)	B(SD)	B(SD)
PCCI (Struggle)	0.477 (0.059)***	0.245 (0.060)***				
PCCI (Lack of Autonomy)			0.095 (0.065)	0.124 (0.052)*		
PCCI (Lack of Social Support)					0.105 (0.065)	0.087 (0.053)
Work–Family Conflict (WFC)		0.363 (0.061)***		0.471 (0.055)***		0.474 (0.055)***
*N*	*222*	*222*	*222*	*222*	*222*	*222*
*R2*	*0.330*	*0.379*	*0.134*	*0.348*	*0.134*	*0.339*

*** *p* < 0.001;

** *p* < 0.001;

* *p* < 0.05;

^ *p* < 0.10.

**Table 4b:** Hayes mediation analysis on parental burnout (Y) with Work–Family Conflict (M) and PCCI (X; including all controls, results not shown) at Time-2 (2022).

	Time-2 (2022)							
	Work–Family Conflict	Parental Burnout	Work–Family Conflict	Parental Burnout	Work–Family Conflict	Parental Burnout	Work–Family Conflict	Parental Burnout
	Model 1		Model 2		Model 3		Model 4	
	B (SD)	B(SD)	B (SD)	B(SD)	B (SD)	B(SD)	B (SD)	B(SD)
PCCI (Struggle)	0.369 (0.091)***	0.128 (0.085)						
PCCI (Lack of Autonomy)			0.193 (0.102)	−0.016 (0.083)				
PCCI (Lack of Social Support)					0.082 (0.109)	−0.012 (0.085)		
Work–Family Conflict (WFC)		0.412 (0.098)***		0.478 (0.092)***		0.476 (0.091)***		0.477 (0.088)***
VIPM							−0.011 (0.084)	0.127 (0.065)^
*N*	*83*	*83*	*83*	*83*	*83*	*83*		
*R2*	*0.298*	*0.340*	*0.182*	*0.319*	*0.149*	*0.319*		

*** *p* < 0.001;

** *p* < 0.001;

* *p* < 0.05;

^ *p* < 0.10.

**Figure 4:**
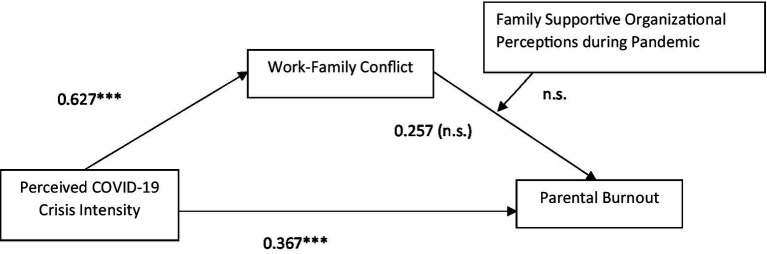
Moderation model (Time-1, 2021).

**Figure 5:**
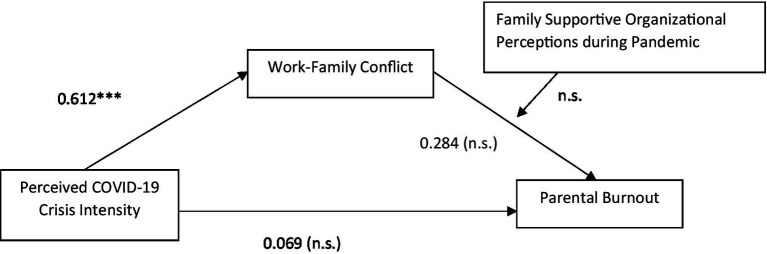
Moderation model (Time-2, 2022).

Hayes Process Model 14 was applied to test the third hypothesis. Hypothesis 3 stated that perceived support from the organization buffers the impact of work–family conflict on parental burnout such that the positive relationship is weaker for those with a perception of positive organizational support than for those perceiving a negative supporting attitude from the organization. Results, as presented in Figures 4, 5, do not show any significant moderating effect on either time point.

Our last hypothesis, 2a, expected that work–family conflict could also mediate the relationship between family tensions toward vaccination and infection prevention (VIPM) and parental burnout. As shown in model 4 of Tables 4, although family tensions and work–family conflict both contribute to parental burnout, there is no mediation effect, because there is no relationship between family tensions and work–family conflict. Furthermore, the relationship between VIPM and parental burnout was only borderline significant.

## Discussion

The first aim of this study was to examine whether and to what extent the perceived intensity of the Covid-19 crisis influences the emergence of parental burnout through work–family conflict. As Mikolajczak and Roskam (2018) suggested that parental burnout may be a consequence of a chronic imbalance of risks over resources, it was hypothesized that protracted exposure to stressors, such as the continuous unpredictable changes in work conditions due to the pandemic, and the decrease in resources, such as one’s usual social support, could lead parents to a state of burnout (cf. Maslach and Leiter, 2016). Accordingly, the present study showed that those who have stronger perceptions of the pandemic intensity indeed experience stronger symptoms of parental burnout both in 2021 and in 2022. Furthermore, higher levels of pandemic perceived intensity were found to be associated with greater levels of work–family conflict, which is in turn related to parental burnout, in both subsequent years of the pandemic. These findings further substantiate and expand Greenhaus and Beutell (1985) theorization of work–family conflict, which suggests that an increase in demands creates or enhances a role conflict between the two spheres. In previous research such demands may stem from the work or the family domain, in our current study demands in both the work and the family domain increased as a result from the pandemic, causing a stronger interference. The relationship between perceived intensity of the Covid-19 crisis and parental burnout was fully mediated by work–family conflict in 2022, yet partially mediated by work–family conflict in 2021. We believe that it was partially mediated in 2021 as apart from the perceived intensity of the Covid-19 crisis to be an antecedent of work–family conflict and subsequently parental burnout, the novelty, unpredictability, threat and lack of control characteristic of the pandemic in 2021 may have led to parental burnout through anxiety and psychological distress. In 2022, much more was known about the virus, the different variants and about treatment of patients. Moreover, a larger share of the population in 2022 was vaccinated or had build-up some immunity.

Interestingly, women were found to experience more parental burnout than men. This is consistent with Roskam et al. (2018) and Roskam and Mikolajczak (2020) outcomes of parental burnout identification: according to their findings the percentage of women in burnout or at high risk was higher than the percentage of fathers in the same conditions. Gender discrepancies were also found in the levels of perceived crisis intensity and in work–family conflict. Consistent with these findings, both Zamarro and Prados (2021) and Shockley et al. (2021) reported that the Covid-19 pandemic disproportionately affected working mothers in terms of childcare load. A study conducted in Italy during the lockdown also reported a gender imbalance in vulnerability to stress, with mothers presenting higher levels of psychological distress than fathers (Marchetti et al., 2020). The authors argued that reasons for this can be traced within the Italian culture, which still holds women, even when involved in professional work, as the ones most responsible for caregiving and for taking care of the household. During the pandemic, children were often obliged to homeschool, and extra-curricular activities were limited or cancelled. Therefore, women may have suffered higher levels of stress and work–family conflict due to the additional pressure exerted by the need of managing children’s care, leisure and homeschooling for days in a row. Additionally, visits and social gatherings were often restrained, and elderly people, identified as the most vulnerable to the Covid-19 disease, were strongly advised to avoid social contacts. This often resulted in a decrease in support coming from grandparents or other members of the social group in the care of children and the household (Cantillon et al., 2021). Consistently, the results of this study found that the time dedicated to childcare after the beginning of the pandemic has increased especially for women, and, in turn, this increase corresponded to higher levels of work–family conflict and parental burnout in 2021. The difference between men and women in time spent on children was no longer significant in 2022, likely related to the opening of schools in 2022. The literature offers wide evidence of how the Covid-19 pandemic enlarged the gender gap in terms of stress (Kowal et al., 2020), income, employment (Kristal and Yaish, 2020) and job satisfaction (Feng and Savani, 2020), the present study further contributes to the research on gender inequality.

The present study also investigated which aspects of pandemic-related changes were more strongly related to parental burnout and work–family conflict. The analyses showed that the elements that were most associated with parental burnout were those pertaining to the PCCI dimension “Struggle and confusion,” which consisted of the fatigue due to higher workload, new work tasks, disfavored methods, contradictory instructions, confusing expectations and unfamiliar tools (tech stress, see also Ghislieri et al., 2021) to perform one’s job. These findings show that strain experienced in one domain (i.e., work) is not only related to the risk of burnout in that same domain (job burnout), as suggested by Bianchi et al. (2014), but it may also impact other domains (i.e., family). This is widely conveyed in the literature concerning spillover from work to family and, vice versa, from family to work. Almost 10 years ago, Berkowsky (2013) advocated that the increasing use of Information Technologies and of working-from-home arrangements leads to an escalating blurriness in work-family boundaries, which in turn enhances the risk for negative spillover from one to the other domain. With the outbreak of the Covid-19 pandemic, telework and the use of IT escalated dramatically, thus probably intensifying negative spillover. In accordance with this, the present study’s results also showed that the first dimension of PCCI, entailing struggle and confusion, was related to work–family conflict.

We did not find an effect of the subscales ‘lack of autonomy’ or ‘lack of social support’ with either parental burnout or work–family conflict. Warr’s (1994) vitamin model offers an interesting explanation. The author argues that autonomy has a curvilinear relationship with wellbeing. While a mild amount of autonomy is a job resource that positively influences performance, health and motivation (Bakker and Demerouti, 2007), too much autonomy can be detrimental for wellbeing, as complex decision making and constant taking of responsibility can lead to an overload of strain. Bredehoeft et al. (2015) consider autonomy as a job demand, as it can entail great psychological costs and emotional exhaustion (Dettmers and Bredehöft, 2020). Connecting this to the present findings, it is conceivable that for some subjects the higher levels of autonomy (often implied by telework) may have been a resource, while for some others they may have been a source of strain. At the same time, the lack of autonomy, perhaps connected with new strict regulations on the workplace, might have been demanding and stressful for some, but reassuring and beneficial for others. In the data of the present study, no curvilinear relationship in 2021 nor 2022 was found between the lack of autonomy and parental burnout. This could be due to the fact that parental burnout is not necessarily similar to the reversed construct of wellbeing (which is the construct for which the curvilinear relationship was hypothesized by Warr, 1994), or, alternatively, to the fact that autonomy may have positive or negative effects depending on individual dispositions and circumstances. Further research could thus investigate the possible reasons behind the unclarity around the role played by autonomy, and whether its benefits are correlated with individual preference, specific beliefs or certain personality styles. A few studies examined the relationship between autonomy and types of personality, such as Conscientiousness or Extraversion (Barrick and Mount, 1993; Gellatly and Irving, 2001); however, more elaboration is needed on the topic. Since working from home is becoming a more and more common arrangement (Eurofound, 2020), and since it generally implies an increase in autonomy (Gajendran and Harrison, 2007; Weinert et al., 2015), it would be of great advantage for organizations to understand for whom and to what extent freedom and autonomy are to be considered beneficial for wellbeing and productivity. As a final contribution to understanding the impact of the Covid-19 pandemic, we examined tensions related to vaccination and infection precaution measures in relation to parental burnout. Consistent with Cusinato et al. (2020) study among Italian parents that lockdown measures and changes in daily routines negatively affected parents’ psychological wellbeing, our study found an indication that tensions related to vaccination and infection precaution measures were related to parental burnout (borderline significant for this sample).

The present research also explored the role of family supportive organizational perceptions during the pandemic. This concept entailed the existence of organizational practices and values that support employees’ general wellbeing and work-family balance. While it was expected that family supportive organizational perceptions would reduce the effect of work-family conflict on parental burnout, this was not demonstrated by our results. However, logically and understandably, it was discovered that family supportive organizational perceptions itself negatively related to the perceived intensity of the pandemic-related changes. *De facto*, supportive organizational practices and values such as caring for employees’ wellbeing, allowing for flexible arrangements, recognizing efforts, considering personal goals and values, accepting and valorizing employees’ private needs may themselves be the reasons why employees may have experienced the pandemic impact as less intense. Similar conclusions were drawn by Fiksenbaum et al. (2006), who demonstrated that higher levels of organizational support predicted lower perceived SARS threat, emotional exhaustion, and state anger in Canadian nurses. It would be interesting to investigate whether organizational support is perceived differently based on gender, and subsequently, if it has a different impact on the perception of the crisis intensity.

Practically speaking, as family supportive organizational perceptions was shown to be associated with lower levels of perceived pandemic intensity, lower levels of work–family conflict and, consequently, fewer symptoms of parental burnout, organizations should redouble their efforts in ensuring support to their employees. Attempts in this direction will serve in soothing the negative long-term changes brought by Covid-19 or by other new stressors and complex job demands that may arise for other reasons, such as economic crises or other disruptive changes. Family-supportive policies and flexible arrangements could prove to be effective resources that can buffer the risk of experiencing high levels of work–family conflict and parental burnout. Gigauri (2020) adds that creating a culture that supports the employees’ physical and psychological wellbeing represents a strategic organizational solution to face the pandemic crisis. More generally, the positive impact of organizational supportive policies and practices as well as a family supportive climate in the organization on employees’ wellbeing can ensure a more sustainable and effective workforce in the long-term (Kossek et al., 2014).

Furthermore, as mothers seem to be suffering more than fathers from the pandemic probably due to the imbalanced division of care and household responsibilities, efforts should be made both on the organizational level and the societal level to incentivize men to share these responsibilities with their partner. Work-life balance programs addressing men, such as paternity and parental leave with income substitution for fathers, seem indeed to be a crucial step toward gender equality, because only when men are given equal opportunities to be caregivers, only when they are accounted for caregiving, will the burden of caregiving responsibilities may not automatically fall onto women (Sweet, 2012; Levs, 2019; Ankiilu, 2021).

## Limitations and future research

This research was conducted 1 and 2 year after the outbreak of the Covid-19 pandemic in Italy. Data were gathered in March/April 2021 and 2022, when the Coronavirus Disease infection rates were growing toward a third peak and plateauing in cases after the fourth infection peak, (WHO, 2022). In Italy, in March and April 2021, movement and encounters were restricted, most commercial activities were interrupted and all schools only used distance learning (Mezza Italia, 2021; Mazzuca, 2022). In March and April 2022 most schools were open, although with strict safety precautions, workplaces, public transport and sports facilities were open when in the possession of valid vaccination passports and with infection precaution measures instilled. This means that data were obtained in particular fractions of the entire pandemic, in 2021 when the harshest conditions were in place and in 2022 at a time when society was gradually opening, yet accompanied with societal polarization on vaccination and infection precaution measures. On the one hand this could be seen as a strong point of the study, as it considered stress levels at their peak in 2021 and lessening in 2022. On the other hand, it could be seen as a limitation, as results cannot be compared to a pre-pandemic moment in time, nor to the situation where Covid-19 has become more or less endemic. Arguably, in other periods of the pandemic year, for example when children were allowed to physically go to school, parents may have carried less burdens concerning childcare and homeschooling, and thus may have experienced less work–family conflict and lower levels of stress. To what extent a situation in which Covid-19 is endemic, where work places and schools may go to and from restriction measures, causing long lasting uncertainty and subsequent parental burnout remains to be seen.

Other critical aspects of this research regard the way of sampling and the study design. Convenience and snowballing sampling were used to enlist respondents for this study. Questionnaires were distributed to friends, family members and acquaintances, who themselves shared the link to the survey with colleagues, or parents having children in the same school. This guaranteed some variety in the sample; however, most of the responses came from northern Italy. Especially in the first part of 2020, northern Italy was the area that was most hit by Covid-19. In most southern regions the virus spread later and restrictions were less intense (Monitoraggio Coronavirus, n.d.). Therefore, it is advisable to be careful in making generalizations to the entire population (Pruchno et al., 2008). Furthermore, sample sizes were relatively small with 222 and 83 respondents at the two time-points. This cautions us to be careful with drawing conclusions. However, since we do find effects it can be very interesting to research the longitudinal effects on parents in existing panels.

A further limitation may be social desirability bias. Lately, fathers and particularly mothers have felt more and more pressure on adhering to the image of the perfect parent: calm, balanced, sensitive, supportive, warm, always available (Daly, 2007). Western social norms prescribe parents, especially mothers, to be fully devoted to childcare and to always put their children’s needs first (Van Engen et al., 2012; Meeussen and Van Laar, 2018). The desire to comply with this ideal may have a twofold outcome. On the one hand, it has been shown that trying to be a perfect parent may increase the susceptibility to parental stress and burnout (Kawamoto et al., 2018; Mikolajczak et al., 2020). On the other hand, however, it could cause biased self-reports, as it might be hard for parents to admit they do not meet this standard (Morsbach and Prinz, 2006; Bornstein et al., 2014). Accordingly, it is plausible that parental burnout was underestimated, especially among women.

A further issue may concern the validity of PCCI: since data preceding the pandemic were non-existent, most of its items implied that respondents compared their present feelings and impressions of their working conditions with their feelings and impressions experienced before the pandemic. It is probable that accuracy in making inferences is limited when thinking retrospectively (Hardt and Rutter, 2004).

Other possible limitations refer to the use of the concept of work–family conflict. While this variable entails negative interference of work with family and family with work, only antecedents regarding the work-domain (and some regarding general isolation) were considered. Further research is advised to utilize an additional variable that specifically considers the pandemic-related changes that occurred within the family, the children’s care and homeschooling. This may offer a wider frame to understand work–family conflict and parental burnout, as it would allow a deeper investigation on their generating factors and their possible combination. Furthermore, we would have liked to be able to control for more demographic variables, such as region, income and other resources at home such as hired helps for household and caregiving. It would have been interesting to see how these would affect the relationships under study.

Additionally, due to the cross-sectional nature of this study, no definitive conclusions about causality can be drawn. For example, while, as theorized, experiencing work–family conflict could lead to a higher degree of parental burnout, it is equally plausible that the experience of high levels of stress and symptoms of burnout causes higher levels of role conflict. Similarly, parents experiencing high levels of work–family conflict may also perceive the intensity of the Covid-19 pandemic more intense. Although we approached all parents of the first data collection in 2022 again, a too small proportion participated in 2022 (*n* = 35) to statistically examine their development in PCCI, work–family conflict and parental burnout. A longitudinal diary study should have been designed in order to get more insight in the causality of the relationships. Such a follow-up study could not only help individuate some degree of causality among variables, but may also provide some further understanding of the long-term effects of the pandemic.

## Conclusion

The objective of the present study was to investigate the relationship between the perceived intensity of the Covid-19 pandemic, characterized by the struggle and confusion, social isolation and impairment of autonomy it induced, and parental burnout, a recently developed construct defined by parental emotional exhaustion, detachment from one’s own children, feelings of low self-efficacy and the acknowledgment of a discrepancy with the previous self in two consecutive years after the first onset of the Covid-19 pandemic. The role of family supportive organizational perceptions was also investigated. Results showed that perceiving a high level of crisis intensity may lead to the emergence of symptoms of parental burnout, and may increase the levels of work–family conflict, which is itself a cause of parental burnout. Unexpectedly, family supportive organizational perceptions did not show any buffering effect in this relationship. However, organizational behaviors and attitudes that support employees’ wellbeing and allow flexible management of work and personal needs were found to directly impact and reduce the perceptions of crisis intensity. This means that organizations, through the implementation of supportive policies and practices and the establishment of a supportive environment, might have the power, and the responsibility, to act upon crisis perceptions and, consequently, upon work–family conflict and parental burnout. Why waste such an opportunity?

## Data availability statement

The datasets presented in this article are not readily available because we did not ask the participants if we could share the data outside our project. Requests to access the datasets should be directed to marloes.vanengen@ru.nl.

## Ethics statement

The studies involving human participants were reviewed and approved by the Ethic Review Board, Tilburg School of Social and Behavioral Sciences, Tilburg University. The patients/participants provided their written informed consent to participate in this study.

## Author contributions

MR wrote first draft of the manuscript, designed questionnaire, collected data at wave 1 and wave 2, cleaned data at wave-1 did analysis for wave 1. ME wrote first draft of the manuscript, revised the manuscript, co-designed questionnaire, and collected data at T1 and T2. SA wrote methods and results section of the manuscript, revised the manuscript, co-designed and programmed wave 2 of the questionnaire, collected data at wave 2, cleaned data, performed analysis for wave 1 and 2. All authors contributed to the article and approved the submitted version.

## Conflict of interest

The authors declare that the research was conducted in the absence of any commercial or financial relationships that could be construed as a potential conflict of interest.

## Publisher’s note

All claims expressed in this article are solely those of the authors and do not necessarily represent those of their affiliated organizations, or those of the publisher, the editors and the reviewers. Any product that may be evaluated in this article, or claim that may be made by its manufacturer, is not guaranteed or endorsed by the publisher.
